# The Next Generation of Risk Assessment Multi-Year Study—Highlights of Findings, Applications to Risk Assessment, and Future Directions

**DOI:** 10.1289/EHP233

**Published:** 2016-04-19

**Authors:** Ila Cote, Melvin E. Andersen, Gerald T. Ankley, Stanley Barone, Linda S. Birnbaum, Kim Boekelheide, Frederic Y. Bois, Lyle D. Burgoon, Weihsueh A. Chiu, Douglas Crawford-Brown, Kevin M. Crofton, Michael DeVito, Robert B. Devlin, Stephen W. Edwards, Kathryn Z. Guyton, Dale Hattis, Richard S. Judson, Derek Knight, Daniel Krewski, Jason Lambert, Elizabeth Anne Maull, Donna Mendrick, Gregory M. Paoli, Chirag Jagdish Patel, Edward J. Perkins, Gerald Poje, Christopher J. Portier, Ivan Rusyn, Paul A. Schulte, Anton Simeonov, Martyn T. Smith, Kristina A. Thayer, Russell S. Thomas, Reuben Thomas, Raymond R. Tice, John J. Vandenberg, Daniel L. Villeneuve, Scott Wesselkamper, Maurice Whelan, Christine Whittaker, Ronald White, Menghang Xia, Carole Yauk, Lauren Zeise, Jay Zhao, Robert S. DeWoskin

**Affiliations:** 1 National Center for Environmental Assessment, U.S. Environmental Protection Agency (EPA), Washington, District of Columbia, USA; 2 ScitoVation, Research Triangle Park, North Carolina, USA; 3 National Health and Environmental Effects Research Laboratory, U.S. EPA, Duluth, Minnesota, USA; 4 Office of Chemical Safety and Pollution Prevention, U.S. EPA, Washington, District of Columbia, USA; 5 National Institute of Environmental Health Sciences, and; 6 National Toxicology Program, National Institutes of Health (NIH), Department of Health and Human Services (DHHS), Research Triangle Park, North Carolina, USA; 7 Department of Pathology and Laboratory Medicine, Brown University, Providence, Rhode Island, USA; 8 Unité Modèles pour l’Écotoxicologie et la Toxicologie, Institut National de l’Environnement Industriel et des Risques, Verneuil en Halatte, France; 9 U.S. Army Engineer Research and Development Center, Research Triangle Park, North Carolina, USA; 10 Department of Veterinary Integrative Biosciences, College of Veterinary Medicine and Biomedical Sciences, Texas A&M University, College Station, Texas, USA; 11 Department of Land Economy, University of Cambridge, Cambridge, England; 12 National Center for Computational Toxicology, and; 13 National Health and Environmental Effects Research Laboratory, U.S. EPA, Research Triangle Park, North Carolina, USA; 14 International Agency for Cancer Research, Lyon, France; 15 George Perkins Marsh Institute, Clark University, Worcester, Massachusetts, USA; 16 European Chemicals Agency, Annankatu, Helsinki, Finland; 17 McLaughlin Centre for Population Health Risk Assessment, University of Ottawa, Ottawa, Ontario, Canada; 18 National Center for Environmental Assessment, U.S. EPA, Cincinnati, Ohio, USA; 19 National Center for Toxicological Research, Food and Drug Administration, Jefferson, Arkansas, USA; 20 Risk Sciences International, Ottawa, Ontario, Canada; 21 Department of Biomedical Informatics, Harvard Medical School, Boston, Massachusetts, USA; 22 U.S. Army Engineer Research and Development Center, Vicksburg, Mississippi, USA; 23 Grant Consulting Group, Washington, District of Columbia, USA; 24 Environmental Defense Fund, Washington, District of Columbia, USA; 25 Education and Information Division, National Institute for Occupational Safety and Health, Centers for Disease Control and Prevention, Cincinnati, Ohio, USA; 26 National Center for Advancing Translational Sciences, NIH, DHHS, Bethesda, Maryland, USA; 27 Division of Environmental Health Sciences, School of Public Health, University of California, Berkeley, Berkeley, California, USA; 28 Gladstone Institutes, University of California, San Francisco, San Francisco, California, USA; 29 Systems Toxicology Unit, European Commission Joint Research Centre, Ispra, Italy; 30 Center for Effective Government, Washington, District of Columbia, USA; 31 Environmental Health Science and Research Bureau, Health Canada, Ottawa, Ontario, Canada; 32 Office of Environmental Health Hazard Assessment, California EPA, Oakland, California, USA

## Abstract

**Background:**

The Next Generation (NexGen) of Risk Assessment effort is a multi-year collaboration among several organizations evaluating new, potentially more efficient molecular, computational, and systems biology approaches to risk assessment. This article summarizes our findings, suggests applications to risk assessment, and identifies strategic research directions.

**Objective:**

Our specific objectives were to test whether advanced biological data and methods could better inform our understanding of public health risks posed by environmental exposures.

**Methods:**

New data and methods were applied and evaluated for use in hazard identification and dose–response assessment. Biomarkers of exposure and effect, and risk characterization were also examined. Consideration was given to various decision contexts with increasing regulatory and public health impacts. Data types included transcriptomics, genomics, and proteomics. Methods included molecular epidemiology and clinical studies, bioinformatic knowledge mining, pathway and network analyses, short-duration in vivo and in vitro bioassays, and quantitative structure activity relationship modeling.

**Discussion:**

NexGen has advanced our ability to apply new science by more rapidly identifying chemicals and exposures of potential concern, helping characterize mechanisms of action that influence conclusions about causality, exposure–response relationships, susceptibility and cumulative risk, and by elucidating new biomarkers of exposure and effects. Additionally, NexGen has fostered extensive discussion among risk scientists and managers and improved confidence in interpreting and applying new data streams.

**Conclusions:**

While considerable uncertainties remain, thoughtful application of new knowledge to risk assessment appears reasonable for augmenting major scope assessments, forming the basis for or augmenting limited scope assessments, and for prioritization and screening of very data limited chemicals.

**Citation:**

Cote I, Andersen ME, Ankley GT, Barone S, Birnbaum LS, Boekelheide K, Bois FY, Burgoon LD, Chiu WA, Crawford-Brown D, Crofton KM, DeVito M, Devlin RB, Edwards SW, Guyton KZ, Hattis D, Judson RS, Knight D, Krewski D, Lambert J, Maull EA, Mendrick D, Paoli GM, Patel CJ, Perkins EJ, Poje G, Portier CJ, Rusyn I, Schulte PA, Simeonov A, Smith MT, Thayer KA, Thomas RS, Thomas R, Tice RR, Vandenberg JJ, Villeneuve DL, Wesselkamper S, Whelan M, Whittaker C, White R, Xia M, Yauk C, Zeise L, Zhao J, DeWoskin RS. 2016. The Next Generation of Risk Assessment multiyear study—highlights of findings, applications to risk assessment, and future directions. Environ Health Perspect 124:1671–1682; http://dx.doi.org/10.1289/EHP233

## Introduction

### Background

Advances in molecular and cell biology provide new insights into the etiology of human disease, largely by evaluating molecular events that influence cell function and interactions (Audouze et al. 2013; Hood and Tian 2012; McCullough et al. 2014, 2016; McHale et al. 2012; Thomas R. et al. 2014). High-throughput and high-content (HT/HC) assays and robotic implementation are generating data streams at unprecedented speeds. Computational tools, automated analytical methods (bioinformatics), and systems biology approaches are being developed to organize and interpret the information (Attene-Ramos et al. 2013, 2015; U.S. EPA 2016; Freitas et al. 2014; Hsu et al. 2014; Huang et al. 2014, 2016; Judson et al. 2011, 2012, 2013, 2014, 2015). The National Library of Medicine databases, Tox21 (Toxicity Testing in the 21st Century), and ToxCast™ (Toxicity ForeCaster) are among the efforts to compile, organize, manage, and store these data to better understand determinants of population health (U.S. EPA 2016; Krewski et al. 2014; NRC 2009) and to help answer such questions as: Which chemicals are environmentally better choices in commerce? Why do individuals and specific subpopulations respond differently to chemical exposures? What happens when people are exposed to low levels of multiple chemicals? How do factors like socioeconomic status and pre-existing illness influence public health risk? How might evaluating and applying these data, methods, and models support environmental health decisions?

A revolution in molecular, computational, and systems biology has occurred over the past 25 years, providing dramatic insights into the causation of disease. This new science, however, has not been extensively incorporated into environmental health risk assessment, although much related research is occurring. To evaluate how new data types and approaches can inform environmental health risk assessments, the U.S. Environmental Protection Agency (EPA) collaborated with several U.S. and international agencies and organizations (see Table S1). We considered the state of science and developed illustrative prototypes (case studies) demonstrating various approaches that investigators could apply to different risk management problems. Our goal was to provide examples that would promote discussion in the risk assessment, risk management, and stakeholder communities and that would facilitate the transition from strategy to practical application.

In this article, we summarize the results of more than 40 separate publications resulting from our collaborative efforts, along with a few key papers by other authors; identify potential application to risk assessment; and articulate strategic research directions. A detailed report of our efforts with an extensive review of the general literature (~ 400 references) is also available (U.S. EPA 2014). Toxicity testing and risk assessment are anticipated to benefit from these advances (Krewski et al. 2014; NRC 2007).

### Objectives

Our specific objectives were to test whether new data sources and risk assessment methods would help *a*) identify specific patterns of molecular events that are associated with impacts of chemical exposures (hazard identification); *b*) characterize exposure–dose within the range of environmental exposures (dose–response); *c*) inform risk factors such as genomic variants, chemical and nonchemical stressor co-exposures (risk modifiers); and *d*) improve indicators of adverse health effects and chemical potency (toxicity surrogates). We also considered how new types of assessments might address differing risk management needs or risk context and help develop decision rules for integrating and applying the available data.

## Methods

We applied and evaluated diverse types of data and methods to determine if, and how, advanced biological data would better inform risk assessments.

### Preparation for Prototype Development

To establish the foundation for this effort, we *a*) worked with the U.S. Environmental Protection Agency (EPA) risk managers to identify research needs and develop a strategy for the overall approach (Cote et al. 2012); *b*) consulted with experts on the concepts for the prototypes (U.S. EPA 2011a); *c*) held a stakeholder conference to inform the public about upcoming activities and to solicit advice (U.S. EPA 2011b); and *d*) developed a framework articulating the guiding principles for NexGen (Krewski et al. 2014).

### Risk Assessments Targeted to Various Decision Contexts

**Figure 1:**
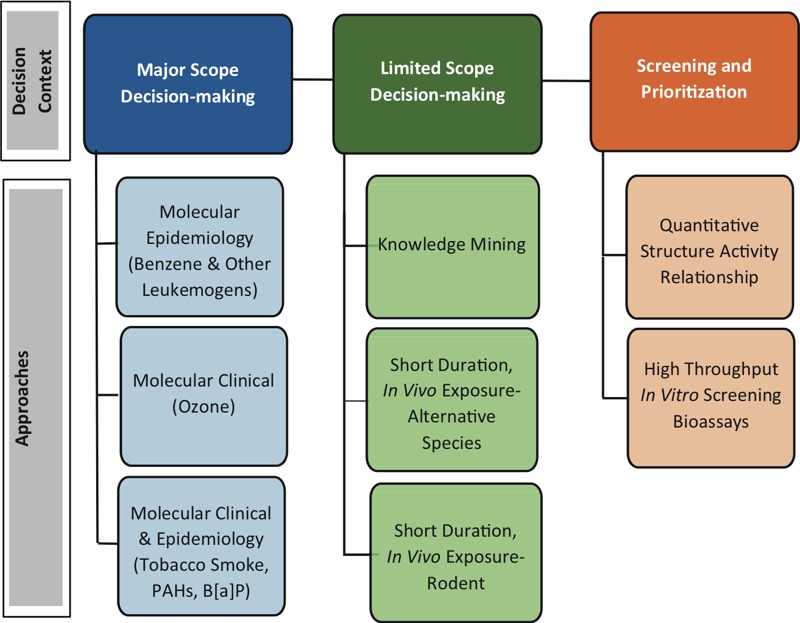
Three broad decision-context categories are shown across the top (white type); the eight “fit-for-purpose” prototypes developed for this effort are shown in black type. From left to right in Figure 1, the amount of traditional toxicological data available for assessment (e.g., *in vivo* rodent toxicity data, epidemiology data) and the confidence in the assessment conclusions decrease, but the number of chemicals that can be evaluated increases markedly. Note: B[a]P, benzo[*a*]pyrene; PAHs, polycyclic aromatic hydrocarbons.

We developed eight prototypes illustrating three decision contexts generally representing environmental challenges risk managers face:

Major scope decisions, usually regulatory decision-making, generally aimed at nationwide exposures and associated risks.
Limited scope decisions, often non-regulatory decision-making, generally aimed at limited exposure, hazard, or data situations.
Chemical screening and prioritization for further testing, research, or assessment or for emergency response (Figure 1). Decision contexts were derived from observation of problems commonly faced by the U.S. EPA (NRC 2009). These generalized decision contexts do not, and are not meant to, capture all decisions or situational nuances risk managers face.

### Study Selection

Establishing systematic review criteria for study selection helps ensure reproducibility, transparency, and scientific acceptability of regulatory actions (McConnell et al. 2014). Our criteria were similar to those used for traditional data (e.g., adequate study design and reporting), augmented with additional criteria specifically applicable to new methodologies (Bourdon-Lacombe et al. 2015; McConnell et al. 2014). Rapidly evolving best practices for advanced biology and certain reporting requirements led many initially considered studies to be deemed inadequate for risk assessment purposes (U.S. EPA 2013b, 2014; McConnell et al. 2014).

### The Prototypes

This section provides an overview of the science considered in the prototypes. Table 1 (adapted from Krewski et al. 2014) summarizes tools and techniques evaluated in the prototypes, organized by decision context. While the tools and techniques are categorized here for simplicity, they represent a continuum of methods that can be applied in various combinations to address agency needs. Additional details are provided both in the papers referenced throughout and in U.S. EPA (2014).

**Table 1:** Prototype use of new scientific tools and techniques applied (1) or not applied (0) (adapted from Krewski et al. 2014).

Tools and techniques	Tier 1: screening and prioritization for further testing, research, or assessment	Tier 2: limited-scope environmental problems and assessments	Tier 3: major-scope environmental problems and assessments
Hazard identification and dose–response assessment methods
Quantitative structure activity relationship models	1	1	0
Pathway–network analysis	1	1	1
High-throughput *in vitro* assays	1	1	1
High-content omics assays	0	1	1
Biomarkers of effect	0	1	1
Molecular and genetic population-based studies	0	0	1
Dosimetry and exposure assessment methods
*In vitro* to *in vivo* extrapolation	1	1	0
Pharmacokinetic models and dosimetry	1	1	1
Biomarkers of exposure and effect	0	1	1
Cross-cutting assessment methods
Adverse outcome pathways	1	1	1
Bioinformatics and computational biology	1	1	1
Systems biology	1	1	1
Functional genomics	0	1	1

***Major-scope assessment prototypes***. Three major-scope prototypes explored how toxicogenomic studies of exposed human populations can inform risk assessment:

Characterizing early key events in the biological cascade that results in adverse outcomes.
Identifying and characterizing biomarkers of exposure and effects.
Identifying factors contributing to population variability and susceptibility.
Elucidating lower exposure–response relationship.

These prototypes used chemicals with known outcomes, robust traditional data, and substantial systems biology understanding to determine if new data types could accurately predict known outcomes—essentially proof of concept for use of molecular biology data in risk assessment. In two of the three prototypes, we compared concomitantly collected traditional and new data types. We considered this an important verification step in order to provide us some confidence that new methods could be successfully applied in situations where data are limited. Additionally, we were interested in examples of how new data types could better inform unresolved uncertainties in chemical assessments based on robust traditional data.

We evaluated transcriptomic and epigenomic data (epidemiological and clinical) in the range of environmental exposures for three chemicals: *a*) benzene and other leukemogens (McHale et al. 2011, 2012; Smith et al. 2011; Thomas R et al. 2012, 2013, 2014); *b*) ozone (Duncan et al. 2012; U.S. EPA 2013a; Hatch et al. 2014; McCullough et al. 2014, 2016); and *c*) polycyclic aromatic hydrocarbons (PAHs), including tobacco smoke and benzo[*a*]pyrene (DHHS 2014; U.S. EPA 2013b; IARC 2010; Mattes et al. 2014). We also considered genomic, proteomic, and epigenomic data as available, and molecular animal and *in vitro* data for benzene and B[a]P (U.S. EPA 2013b; French et al. 2015). We evaluated exposures for benzene of < 0.1 to 10 parts per million (ppm) and ozone of 0.5 ppm for 2 hr. We used individual measures of the exposure–dose relationship for benzene and ozone (benzene urinary metabolites (Vermeulen et al. 2004) and heavy oxygen-labeled ozone (^18^O_3_) (Hatch et al. 2014). For PAH exposures, we used self-reported smoking. The PAH–tobacco smoke prototype focused on pathway mining of existing human microarray data from the ArrayExpress (http://www.ebi.ac.uk/) and Gene Expression Omnibus (http://www.ncbi.nlm.nih.gov/geo/). The toxicogenomics data were compared qualitatively and quantitatively to known health outcomes associated with these chemicals, specifically hematotoxicity and leukemia (benzene and other known leukemogens), lung inflammation and injury (ozone), and lung cancer (PAHs). The results of these data-rich comparisons therefore enabled us to draw on a wealth of chemical- and disease-specific data to help characterize associations among upstream molecular changes, downstream cellular events, and public health outcomes. Thus, the potential role of toxicogenomics in hazard identification and dose–response assessment was explored.

***Limited-scope assessment prototypes.*** These prototypes explored approaches falling between molecular human clinical and epidemiology studies (described in “Major-scope assessment prototypes”) and *in vitro*, HT screening bioassays (described in “Screening and prioritization prototypes”) in terms of confidence in the data to characterize public health risks, resources expended to collect data, and the number of chemicals that can be evaluated in a given period. We considered three approaches to limited-scope assessment:

Knowledge mining of large health databases [focusing on human tissue biomonitoring and diabetes data from NHANES (National Health and Nutrition Examination Survey)] (Bell and Edwards 2015; DeWoskin et al. 2014; U.S. EPA 2014; Patel et al. 2012, 2013a; Thayer et al. 2012).
Short-duration *in vivo* exposures using alternative (nonmammalian) species (focusing on the thyroid hormone disruptor mechanism and zebrafish developmental outcomes for several hundred chemicals) (Padilla et al. 2012; Perkins et al. 2013; Sipes et al. 2011a, 2011b; Thienpont et al. 2011; Villeneuve et al. 2014).

Short-duration *in vivo* exposure rodent studies that correlated transcriptomic alterations with cancer and noncancer outcomes as determined in traditional bioassays (Thomas RS et al. 2012a, 2013a, 2013c).

Advantages of the limited-scope approaches compared to HT *in vitro* approaches include intact metabolism and intact cell and tissue interactions and the potential to measure adverse health outcomes, including complex outcomes such as altered behavior and development.

***Screening and prioritization prototypes.*** The two screening and prioritization prototypes are *a*) quantitative structure activity relationship (QSAR) models and use of analogous chemicals to expand available information (also called “read-across”) (Golbraikh et al. 2012; NAFTA Technical Working Group for Pesticides 2012; OECD 2016a; Politi et al. 2014; Wang et al. 2011, 2012a); and *b*) *in vitro* cell-based and biochemical-based (including enzymatic and ligand-binding) HT screening assays [focusing on evaluating thyroid hormone disruptors (Cox et al. 2014; Rotroff et al. 2013; Sipes et al. 2011a; Judson et al. 2010)]. Of note, although QSAR and *in vitro* assays are illustrated separately here, they often are used most effectively in combination. The U.S. EPA’s ToxCast™ program (Judson et al. 2010, 2011, 2012, 2013, 2014; Kavlock et al. 2012) and the multi-agency collaborative Tox21 program (Attene-Ramos et al. 2013, 2015; Freitas et al. 2014; Hsu et al. 2014; Huang et al. 2014; Tice et al. 2013) provide more information on these approaches. Virtual tissue modeling (DeWoskin et al. 2014; Knudsen and DeWoskin 2011; Knudsen et al. 2013, 2015) and toxicokinetic approaches (Wambaugh et al. 2015; Wetmore et al. 2012, 2013) also are discussed.

***Examining human variability in responses.*** The data to evaluate variability and susceptibility are usually scant. We evaluated several data types to inform this issue:

Adverse outcome networks (AON) to identify mechanistic commonalties among leukemogens and lifestyle factors (diet and stress) that alter leukemia risks (U.S. EPA 2014; IARC 2012; Smith et al. 2011).
Altered disease incidence in subpopulations having specific genetic polymorphisms (U.S. EPA 2014).
Data for *in vitro* cells that retain an asthma phenotype in ozone studies (Duncan et al. 2012).
Correlated measurements of phenotypic differences among diverse subpopulations with different incidences of given exposures [tissue biomonitoring using NHANES (U.S. EPA 2014; Patel et al. 2012, 2013a)].
HT *in vitro* data from cell lines with different genetic backgrounds from the 1,000 genomes effort (Abdo et al. 2015a, 2015b; Attene-Ramos et al. 2015; Lock et al. 2012; O’Shea et al. 2011).
Computational modeling in which variability in parameter values is simulated for differences among subpopulations (Knudsen and DeWoskin 2011; Shah and Wambaugh 2010).

Adverse outcome networks are conceptual mechanistic models that combine key events and adverse outcome pathways (AOP) into networks associated with specific diseases and disorders [see Zeise et al. (2013) and NRC (2011) for further details on examining human variability].

## Results and Discussion

The NexGen prototypes help us to better understand and apply emerging science in a transparent and scientifically robust manner to environmental health risk assessment. Additionally, these prototypes help realize the National Research Council’s vision embodied in *Toxicity Testing in the 21st Century: A Vision and a Strategy* (NRC 2007; Krewski et al. 2011). Since this report was published, toxicity testing and risk assessment has continued shifting from the traditional, almost exclusive, use of animal data to using the new approaches the prototypes demonstrate (Adeleye et al. 2015; Abdo et al. 2015a, 2015b; Attene-Ramos et al. 2015; Bourdon-Lacombe et al. 2015; EC 2016, EC and JRC 2015; ECHA 2016a, 2016b; U.S. EPA 2015a, 2015b; Huang et al. 2016; JRC 2016; Mansouri et al. 2016; OECD 2016a, 2016b; Wambaugh et al. 2015). The new approaches consider a broader data array, foster mechanistic understanding of adverse effects, and move toward replacing uncertainty factors and extrapolations with data-derived probability distributions.

In each decision context category, new methods and data types were identified that could help inform assessment efforts. Methods illustrated in the screening and prioritization (Tier 1) and limited-scope (Tier 2) prototypes originally were designed for qualitative evaluation of chemicals. New and integrated approaches, however, are being developed to estimate relative potencies and more rapid quantitative toxicity values for use in certain decision contexts.

We used AOP and AON extensively to organize and interpret data for most of the prototypes and regard them as critical for linking molecular events to apical outcomes. The AOP–AON concept has gained considerable traction since it was first introduced (Ankley et al. 2010; Davis et al. 2015; Garcia-Reyero 2015; Geer et al. 2010; Tollefsen et al. 2014; Vinken 2013). We use the terms AOP and AONs throughput this article as they are commonly used by many U.S. and European agencies (OECD 2013).

Data quality and reporting are always critically important. Our data searches identified many published studies that we could not use because the data or the reporting was not sufficient for use in health risk assessment (e.g., does not meet minimum standards for study design or reporting) (U.S. EPA 2014; McConnell et al. 2014). This situation derives from the lag between establishing best practice criteria and full implementation in the research community, and inconsistent application of criteria for data quality and reporting (U.S. EPA 2014; McConnell et al. 2014).

Integrating the available data into a coherent analysis is also a challenge. Table S2 presents the evidence integration framework used for the prototypes. The framework focuses on evaluating and integrating evidence and drawing conclusions based on inferences drawn from new data types. To our knowledge this illustrative framework is the most complete illustration of using a new data type in a variety of assessment situations. More limited examples of evidence integration using new approaches include *a*) the International Agency for Research on Cancer’s determination of a likely causal link between benzene exposures and lymphoma based on molecular mechanisms data (IARC 2012); *b*) the U.S. EPA’s cumulative risk evaluation of relatively uncharacterized conazole fungicides based on molecular mechanisms data (U.S. EPA 2011d); *c*) the U.S. EPA’s use of toxicogenomic data in the Endocrine Disruptor Screening Program (EDSP) (Mansouri et al. 2016; U.S. EPA 2011c); *d*) OECD’s guidance on use of adverse outcome pathways in toxicity evaluations (OECD 2013); and *e*) OECDs guidance on the use of quantitative structure activity data to evaluate relative toxicity, and other activities on molecular screening and toxicogenomics (OECD 2016a, 2016b).

### Major-Scope Assessment Prototypes (Tier 3)

We designed the Tier 3 prototypes to determine whether new data types could provide results comparable to robust traditional data. We also evaluated whether new data types could add to information robust traditional data sets provide. Support for this hypothesis and several sources of variability are given below (U.S. EPA 2013a, 2014; Esposito et al. 2014; Hatch et al. 2014; McCullough et al. 2014; McHale et al. 2011, 2012; Smith 2010; Smith et al. 2011; Thomas R et al. 2014). Highlights from the prototypes include:

AONs, once verified for accuracy, are useful in predicting specific hazards [e.g., benzene and other known leukemogens (hematotoxicity) (U.S. EPA 2014; IARC 2010; McHale et al. 2012; Smith 2010; Smith et al. 2011; Thomas R et al. 2012, 2014), ozone (lung inflammation and injury) (U.S. EPA 2013a, 2014; McCullough et al. 2014, 2016; Wu et al. 2015), and PAHs (lung cancer) (U.S. EPA 2013b, 2014; Mattes et al. 2014)].
Related chemical and nonchemical stressors (known to cause or exacerbate the same adverse health outcome) were shown to perturb various pathways within the same disease associated network, but do not always affect the same expressed genes or pathway (U.S. EPA 2014). Hence, overly simplistic descriptions of AOPs could miss the potential for network-level interactions. Evidence for a causal relationship between a specific AOP and adverse effects includes pharmacologic intervention to block identified pathway changes, use of knock-in and knock-out models, or identification of pathway polymorphisms and concomitant amelioration of severity or incidence of the specified adverse outcomes (U.S. EPA 2014; French et al. 2015; Hatzimichael and Crook 2013; Kasahara et al. 2015; McCullough et al. 2014; McHale et al. 2012; Smith 2010; Thomas R et al. 2014; Wu et al. 2015).
Less well-studied chemicals inducing the same AOP or AON could be of concern for concomitant health outcomes. Conversely, lack of an apparent mechanistic link to an adverse outcome might justify downgrading questionable *in vivo* data. Thus, network-level knowledge often is highly valuable to understand causal mechanisms, help integrate evidence, assess potential hazards of well-studied chemicals, provide a basis for cumulative assessment by grouping chemical and nonchemical stressors according to their common AOP network, and evaluate mechanisms underlying human susceptibility (e.g., genetic differences) (Bell et al. 2016; Carter et al. 2013; Ideker and Krogan 2012; Kleinstreuer et al. 2016; Schadt and Björkegren 2012; Zhang et al. 2014; Smith et al. 2016).
Biomarkers appropriately anchored to *in vivo* results can help elucidate exposure–dose–response relationships. Thomas R et al. (2014), extending the work of McHale et al. (2011), best illustrates use of molecular biomarkers to potentially predict public health risks. They reported dose-dependent effects of benzene exposure on gene expression and biochemical pathways, using transcriptome profiling of peripheral blood mononuclear cells, in people (< 1 ppm to > 10 ppm). Benzene exposures were estimated by urinary benzene levels. They estimated dose–response of gene expression in acute myeloid leukemia (AML) and related pathways. Responses at or below 0.1 ppm benzene were observed for altered expression of AML pathway genes and CYP2E1. Together, these data show that benzene alters disease-relevant pathways and genes in a dose-dependent manner. It should be noted that while benzene is considered a known hematotoxicant and leukemogen, the benzene exposed population from which the toxicogenomic biomarkers were characterized at this time only show hematotoxicity (U.S. EPA 2014). The leukemia lag time is such that additional follow-up will be required to demonstrate if the toxicogenomic signature is predictive of leukemia in the same individuals. Understanding the quantitative relationship of any biomarker to exposure and effect requires substantial study. A most promising application of biomarkers, however, is the ability to measure events of interest directly in environmentally exposed humans—an application revolutionizing epidemiology.
For benzene, ozone, and theoretically for PAHs, we demonstrated that multiple AOPs developed and progressed with increasing exposures (U.S. EPA 2014). With benzene, gene and pathway alterations associated with altered proliferation and differentiation, DNA-repair and immune function, among others, were discussed; impaired immune function was shown to occur at all exposure levels evaluated (from < 0.1 ppm to 10 ppm) (French et al. 2015; Thomas R et al. 2014). At higher concentrations, molecular pathways and effects characteristic of more severe toxicity (apoptosis and cell death) begin to emerge (French et al. 2015; Thomas R et al. 2014). Data collection over a range of concentrations thus remains essential when evaluating new data types. Additionally, limited time-course post-exposure data were available for ozone; various adverse outcomes involved in lung injury progressed after exposure, demonstrating the potential dynamic nature of underlying mechanisms (U.S. EPA 2013a; McCullough et al. 2014, 2016).
Chemical exposures resulting in adverse outcomes (e.g., benzene induced leukemia or ozone induced inflammation) appear to share AOP networks with pathologies of unknown origins (e.g., idiopathic or potentially naturally occurring disease) (U.S. EPA 2013a; Hatzimichael and Crook 2013; McCullough et al. 2014, 2016; McHale et al. 2012; Smith 2010; Smith et al. 2011; Thomas R et al. 2014; Wu et al. 2015). This suggests that chemically induced events might add to naturally occurring backgrounds of disease via shared mechanisms (U.S. EPA 2014). As NRC (2009) and Crump et al. (1976) discuss, this observation might have implications for an assumption of low-dose linearity for cancer and noncancer outcomes at the population level.
The prototypes helped characterize experimental and organismic factors influencing data interpretation, including experimental variability resulting from differing exposure concentrations, dosimetry, time courses, experimental techniques, experimental paradigms, cell and tissue types, individual genomic profiles, co-exposures, and lifestages (Ankley and Gray 2013; Bell and Edwards 2015; Cho et al. 2013; U.S. EPA 2014; French et al. 2015; Godderis et al. 2012; Hatch et al. 2014; McCullough et al. 2014; McHale et al. 2014; Mendrick 2011; Perkins et al. 2013; Smith 2010; Smith et al. 2011; Thomas R et al. 2014; Thomas RS et al. 2012b; Tice et al. 2013; Zeise et al. 2013). Identifying causal events without tight control of variability can be difficult even knowing the adverse outcome, reinforcing the importance for careful experimentation and interpretation when potential outcomes are unknown (U.S. EPA 2014).

### Limited-Scope Assessment Prototypes (Tier 2)

We designed the Tier 2 prototypes to evaluate data from knowledge mining, alternative species bioassays, and short-term *in vivo* studies for identifying potential hazards, refining mechanistic understanding, and characterizing the relative potencies of thousands of chemicals more rapidly than possible with traditional methods. Confidence in these data generally ranks between Tier 3 and Tier 1 approaches. Highlights from the prototypes include:

These approaches are faster and less expensive than the molecular human epidemiology studies noted above and traditional chronic animal bioassays. Furthermore, unlike the QSAR models and HT screening data (discussed below), the data from *in vivo* studies are from intact organisms with metabolic function, normal architecture (for various cell and tissue types), and normal cell-cell, tissue-tissue interactions. The data also can be used to study more complex system-level adverse outcomes, such as developmental and neurobehavioral outcomes.
In the data-mining exercises, specific chemical exposures were associated with altered risks for diabetes or prediabetes (e.g., chlorinated organics, heavy metals, selected nutrients) (Bell and Edwards 2015; U.S. EPA 2014; Patel et al. 2012, 2013a, 2013b). We mined exposure data from NHANES human tissue biomonitored levels and NHANES clinically defined incidence. Additional risk factors—multiple chemical exposures and genetic and lifestyle susceptibility traits—were identified (Bell and Edwards 2015; U.S. EPA 2014; Patel et al. 2012, 2013a, 2013b). In one example, 59% of people with high levels of cadmium, lead, and arsenic also had markers for diabetes (U.S. EPA 2014). The data-mining results are generally most suitable for hypothesis generation because the output only identifies associations among events in very large data sets. The availability of biomonitoring data and clinical diagnoses in the same individuals, or understanding of mechanisms, however, is useful in an evidence analysis. Others also have provided traditional and computational data that report an association between chemical exposure and diabetes (Audouze et al. 2013; Dimas et al. 2014; Inadera 2013; Thayer et al. 2012).
Two Tier 2 prototypes demonstrated use of short-duration exposure bioassays in alternative species and mammalian species. We evaluated the results with traditional, molecular, and computational approaches. Collectively, these bioassays successfully identified exposure concentrations associated with transcriptomic changes, AOP–AON alterations and adverse outcomes (Padilla et al. 2012; Perkins et al. 2013; Thomas RS et al. 2011, 2012b, 2013c; Villeneuve et al. 2014). These prototypes provided data on complex mechanistic behaviors, effects of mixtures, and species-to-species similarities and differences, illustrating how these data could be used to evaluate potential hazards and chemical potencies (Ankley and Gray 2013; LaLone et al. 2014; Padilla et al. 2012; Painter et al. 2014; Perkins et al. 2013; Thomas RS et al. 2013b, 2013c).

### Screening and Prioritization Prototypes (Tier 1)

For the first time, new approaches are being used that can evaluate vast numbers of chemicals relatively rapidly. For example, the tens of thousands of chemicals covered by the European Regulation on Registration, Evaluation, Authorisation and Restriction of Chemicals (REACH) legislation are being evaluated using QSAR and new types of bioassays (EC 2016; ECHA 2016a, 2016b; JRC 2016; OECD 2013, 2016a, 2016b). The U.S. Tox21 program is screening approximately 8,500 chemicals using innovative robotic technology and *in vitro* bioassays (Tice et al. 2013). Kavlock et al. (2012) note that “These tools can probe chemical-biological interactions at fundamental levels, focusing on the molecular and cellular pathways that are targets of chemical disruption.” The QSAR models (Goldsmith et al. 2012; Venkatapathy and Wang 2013; Wang et al. 2012a, 2012b) and HT *in vitro* bioassays were used to illustrate the rapid successful screening and prioritization of chemicals (Judson et al. 2013; Kavlock et al. 2012; Kleinstreuer et al. 2014; Rusyn et al. 2012; Sipes et al. 2013; Tice et al. 2013). Additional insights include:

An essential element to evaluating and applying HT data within the risk paradigm is dose characterization. Researchers are developing methods using reverse dosimetry to extrapolate bioactive concentrations in *in vitro* test systems to the comparable doses for *in vivo* exposure to rodents (or other test species) or to humans [*in vitro*-to-*in vivo* extrapolation (IVIVE)] (Abdo et al. 2015a, 2015b; Eduati et al. 2015; Hubal 2009; Rotroff et al. 2010; Wambaugh et al. 2015; Wetmore et al. 2012, 2013). IVIVE extrapolation supports quantitative comparisons of *in vitro* toxicity results with *in vivo* bioassay results for estimating dose–response in human exposures.
QSAR, *in vitro,* and *in silico* methods are proving useful for screening and ranking large numbers of chemicals for further assessment and urgent-response situations where traditional data are lacking (Adeleye et al. 2015; ECHA 2016a, 2016b; Eduati et al. 2015; Judson et al. 2015; Knudsen et al. 2015; NAFTA Technical Working Group on Pesticides 2012; OECD 2016a, 2016b; Ryan et al. 2016; Nishihara et al. 2016). Current estimates of human disease risks based exclusively on QSAR and *in vitro* HT screening generally are too uncertain for many applications (Casey et al. 2015; Cox et al. 2014; EC and JRC 2015; U.S. EPA 2014; Settivari et al. 2015). Recent advances, however, are improving our understanding of these data. Insights into underlying mechanisms of toxicity, and the factors that might contribute to population variability in response to chemical exposure (Abdo et al. 2015a, 2015b; Duncan et al. 2012; Eduati et al. 2015; Lock et al. 2012; O’Shea et al. 2011), are also progressing from these data streams and increasing their utility for understanding risks.

### Caveats Pertaining to Applying New Data Types in Risk Assessment

Exposure and adverse outcomes often can be associated with hundreds to thousands of gene changes, not all of which are causal (Mendrick 2011). Associative data can “suggest” a causal relationship between exposure and adverse health outcomes. Criteria to move from “suggestive” to “likely” causal include meta-analyses of multiple, independent studies yielding similar results; experimental evidence of causative relationships between key events in AOP networks and consequent adverse health outcomes; or combinations of consistent, coherent traditional and new data types. The prototypes demonstrated how different types of evidence in each decision support category might be characterized with respect to establishing causality and evidence integration (U.S. EPA 2014; NRC 2014). Additional caveats are described below. Many of these concerns apply to traditional, as well as new data types.

Cell type, tissue, individual, subpopulation, strain, species, and test system can affect how specific alterations in molecular events manifest as adverse outcomes or disease, even when the molecular signature is the same (Ankley and Gray 2013; Bell and Edwards 2015; Cho et al. 2013; U.S. EPA 2014; French et al. 2015; Godderis et al. 2012; Hatch et al. 2014; McCullough et al. 2014; McHale et al. 2014; Mendrick 2011; Perkins et al. 2013; Smith 2010; Smith et al. 2011; Thomas R et al. 2014; Thomas RS et al. 2012b; Tice et al. 2013; Zeise et al. 2013).

**Figure 2:**
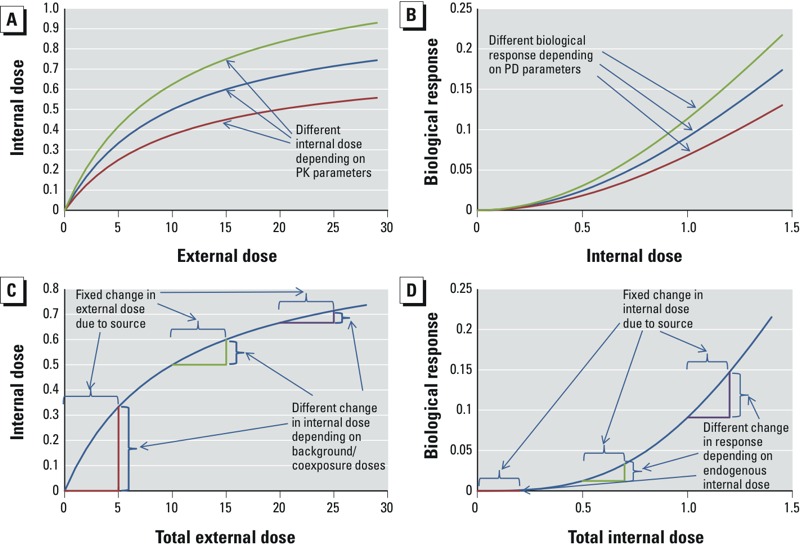
Effects of variability in (*A*) pharmacokinetics (PK), (*B*) pharmacodynamics (PD), (*C*) background and exposures, and (*D*) endogenous concentrations. In (*A*) and (*B*), individuals differ in PK or PD parameters. In (*C*) and (*D*), individuals have different initial baseline conditions (e.g., exposure to sources outside of the risk management decisions context; endogenously produced compounds) (Zeise et al. 2013). Reproduced with permission from *Environmental Health Perspectives*.

This phenomenon likely is due, at least in part, to dosimetry, epigenomic differences, and genomic plasticity, which assessments should consider whenever feasible.
For many chemicals, metabolism is critical to toxicity. That most HT *in vitro* test systems have limited or no metabolic competence should be considered. Although researchers are evaluating various approaches to add or enhance metabolic capability, satisfactory solutions that incorporate metabolism for routine screening of larger numbers of chemicals are not yet available. Consequently, although positive results are informative, negative results should not yet be interpreted as a lack of toxicity.
Molecular profiles can be both dose and time dependent (Knudsen et al. 2013, 2015; McCullough et al. 2014; Perkins et al. 2013; Thomas R et al. 2014; Thienpont et al. 2011). Predicting adverse outcomes based only on “snapshots” of biological events can therefore be challenging. Focusing on profiles associated with environmentally relevant exposures should improve predictions. Some signatures do appear stable over time, however, and might also serve as reliable indicators of chronic outcomes (Thomas RS et al. 2013c).
Gene expression data contain much uncertainty, as messenger RNA expression levels cannot be used to infer protein activity directly. Thus, these data alone could be suitable only for ranking and screening and used in assessments to complement other mechanistic data.
Our current ability to monitor multiple molecular processes (genomics, transcriptomics, proteomics, and epigenomics) in a single study is very limited, primarily due to cost. This hampers biological integration and limits our understanding of how chemicals influence complex biological systems.
A major challenge in using molecular data in risk assessment is how to use the data to improve predictions of adverse effects in humans. For example, how do changes in molecular events affect cells, changes in cells affect tissues and organs, and changes in organs affect the whole body? Researchers are collecting large amounts of HT/HC screening data on molecular-level effects, and the body of information on diseases and disease outcomes is substantial (http://www.ncbi.nlm.nih.gov/geo/; EC 2016; EC and JRC 2015; Huang et al. 2016; Tice et al. 2013). Very sparse chemical-specific data are available, however, on intermediate levels of organization and on the sequence of cellular-level disruption of normal biology to effects at higher organizational levels. Even so, tremendous strides are being made in generating disease-specific information.
Characterizing population response variability among individuals is a major challenge, given the many sources of inherent biological variability (e.g., genetic differences) and extrinsic influences (e.g., lifestyle, poverty, multiple chemical exposures). Each chemical exposure–health outcome pair involves combinations of these sources, and different decision contexts present distinct needs regarding the identification—and extent of characterization—of interindividual variability in the human population (see Figure 2). New approaches to examining variability in responses include *a*) computational modeling, in which variability in parameter values is simulated and differences among subpopulations are explored (Diaz Ochoa et al. 2013; Knudsen and DeWoskin 2011; Knudsen et al. 2015; Shah and Wambaugh 2010); *b*) HT *in vitro* data analysis of cell lines with different genetic backgrounds from the 1000 Genomes effort (Abdo et al. 2015a, 2015b; Eduati et al. 2015; Lock et al. 2012; O’Shea et al. 2011); *c*) human clinical and *in vivo* animal studies in genetically diverse individuals to identify genetic and epigenetic determinants of susceptibility (French et al. 2015; Harrill et al. 2009a, 2009b; McCullough et al. 2016); *d*) comprehensive scanning of gene coding regions in diverse individuals to examine the relationships among environmental exposures, interindividual sequence variation in human genes, and population disease risks (Mortensen and Euling 2013; NIEHS 2015); *e*) genome-wide association studies to uncover genomic loci that might contribute to risk of disease (NHGRI 2015; Wright et al. 2012); and *f*) association studies correlating phenotypic differences among diverse populations with expression patterns for groups of genes based on coexpression (Friend 2013; Patel et al. 2012, 2013a; Weiss et al. 2012). Additionally, understanding of the contribution of epigenomics to disease is the focus of much research (Ghantous et al. 2015).
Verifying toxicity-testing schemes and computational models that are more efficient is essential for using these new data and approaches for risk-based decisions. Central to this effort are a framework and criteria for determining whether the new data types are adequate for various types of decisions. While ultimately different methods and models based on their ability to predict human outcomes, they are also evaluated against their intended purpose. For example, high-throughput methods that can relatively rank thousands to tens of thousands of chemicals, with some certainty, based on their potential toxicity would be deemed extremely successful even though they may not be able to predict the specific health outcome anticipated in humans. Alternatively, methods and models relied upon to support regulation must contribute to the understanding of public health risks. The level of certainty needed in the data varies with their intended use because inaccurate results have increasing consequences and costs as decisions progress from screening, to further testing, to what safe chemical levels are, to what regulatory (or mitigation) actions should be taken (Crawford-Brown 2013). Traditional validation approaches that evaluate conventional assay and testing structures do not adequately address the potential uses of these new data and methods and would require years to implement (Judson et al. 2013). Thus, as the technology for rapid, efficient, robust hazard testing advances, the verification process also must advance to ensure confidence in their use. Clear and transparent articulation of these decision considerations are essential to the acceptance of, and support for, assessment results and in the overall evidence integration. Crawford-Brown (2013) discusses these issues relative to NexGen more extensively.

Based on the lessons learned in the NexGen program and elsewhere, several new types of high- and medium-throughput assessments are being advanced (Casey et al. 2015; ECHA 2016b; U.S. EPA 2014, 2015b; Langley et al. 2015; Perkins et al. 2013; Settivari et al. 2015). Table 2 shows how characteristics of “fit-for-purpose” assessments could be tailored to support three illustrative decision-context categories. The table lists potential uses for NexGen assessments, data sources and types in different assessment categories, exposure paradigms used, incorporation of toxicokinetics, use of traditional data, hazard characterization, potency metrics, inferences drawn about the causal associations between exposures and adverse outcomes, the numbers of chemicals that can be assessed, and the time to conduct any given assessment.

**Table 2:** Possible characteristics of fit-for-purpose assessments matched to illustrative decision-context categories.

Characteristics	Tier 1: screening and prioritization	Tier 2: limited-scope assessments	Tier 3: major-scope assessments
Uses of NexGen assessments	Screening chemicals with no data other than QSAR or HT data. For example, Queuing for research, testing, or assessment Urgent or emergency response	Generally nonregulatory decision-making. For example, Urban air toxics Potential water contaminants Hazardous waste and superfund chemicals Urgent or emergency response	Often regulatory decision-making. For example, National risk assessments Community risk assessment Special problems of national concern
Data sources	EPA databases such as ACToR and ToxCast™; NIH National Center for Biotechnology Information (NCBI) databases, such as BioSystems, Gene Expression Omnibus, Pubchem (http://www.ncbi.nlm.nih.gov/gquery/?term=NCBI)	Large public data and literature repositories [e.g., NIH NCBI PubChem, BioSystems; NHANES; European ArrayExpress (http://www.ebi.ac.uk/)]	All sources of policy-relevant data
New data types (Also uses the data from column to left)	QSAR, HT *in vitro* screening assays, read- across, AOP development	High-content assays, medium-throughput assays, knowledge-mined large data sets, AOP development	Molecular epidemiology, clinical and animal studies, AOP network development
Exposure paradigms of studies considered	*In vitro, in silico*	All relevant	All relevant
Metabolism in test systems	Some to none	Partial to intact	Intact
Incorporation of toxicokinetics	Reverse toxicokinetic models	Reverse toxicokinetics models, biomonitoring	Dosimetry and PK modeling, biomonitoring
Consideration of human variability and susceptibility	*In vitro* methods available	*In vitro* and *in vivo* methods available	*In vivo* methods available
Use of traditional *in vivo* data	*In vitro* assays anchored to pesticide registration and pharmaceutical data	None to limited; especially can be used in AOP development	New data types augment traditional; traditional data currently remain basis for assessment
Hazards	Nonspecific	Nonspecific to identified	Identified
Potency metrics	Relative rankings based on QSAR or HT toxicity values	Relative rankings and toxicity values	Risk distributions, cumulative & community risks
Likely strength of evidence linking exposure to effect	Suggestive to likely	Suggestive to likely	Suggestive to known
Numbers of chemicals that can be assessed	10,000s	100s–1,000s	100s
Time to conduct assessment	Hours–days	Hours–weeks	Days–years
Note: ACToR, Aggregated Computational Toxicology Resource (U.S. EPA); NHANES, National Health and Nutrition Examination Survey; NIH, National Institutes of Health; PK, pharmacokinetic.			

### Research Needs

Enhancing our understanding of complex chemical and biological interactions at various levels of biological organization requires integrating computational research with strategic laboratory studies to advance available models and accelerate application of new data in risk assessment. We suggest focusing on the following specific areas:

Developing reliable, molecular biomarkers and bioindicators that represent a wide variety of chemical exposures and key events of pathogenesis for building confidence in the characterization of key events used to construct an AOP.
Identifying and understanding AOP network interactions among different levels of organization for observed key events (genes, proteins, cells, tissues, organs, individuals, populations, and communities), including characterizing compensatory responses and their prognostic value for different adverse outcomes or disease states.
Collecting data and developing methods for *a*) reverse toxicokinetics to extrapolate concentrations used in cellular and cell-free systems to *in vivo* tissue doses and exposures, *b*) nonaqueous *in vitro* exposure methods for chemicals present as gases or as airborne particles, and *c*) adjusting for intra- and interspecies differences when assessing potential human effects based on nonhuman toxicity data.
Developing approaches for grouping chemical and nonchemical stressors based on common key events within AOPs to enable cumulative risk assessment and consideration of source apportionment with respect to exposures for cumulative risk assessment.
Evaluating individual human variability due to lifestage vulnerabilities, genetic differences, pre-existing disease and exposure, or adaptive and compensatory capabilities and developing techniques to incorporate this variability into population-level risk assessment.

## Conclusions

A revolution in molecular, computational, and systems biology is rapidly advancing our understanding of what causes disease and who becomes affected, and the role of environmental factors on public health. This information is just beginning to result in innovative, more efficient approaches to toxicity testing and risk assessment. This article summarizes recent, multi-organizational efforts to understand and apply emerging science in a transparent and scientifically robust manner to environmental health risk assessment. We anticipate these novel methods will provide a more complete understanding of the biological underpinnings of health risks and, also, methods and data to help evaluate the tens of thousands of unaddressed chemicals in the nation (U.S. EPA 2015c). The overarching challenge to risk assessors is to obtain and interpret sufficient data for quick and efficient assessment to support decisions that protect public health and the environment. The ultimate goal is to develop safer chemicals and to better manage risks to public health and the environment. The prototypes demonstrate how new data can be used to help address these challenges.

The following list presents the ongoing efforts to advance toxicity testing and risk assessment:

Thousands of chemicals, previously having no or very limited traditional data, are being assessed based on similarities in physical–chemical structure to known toxicants (QSAR modeling) and on the results of rapid, robotically conducted *in vitro* bioassays. These evaluations will help prioritize testing, research, and assessment, and responding in emergency response situations.
Hundreds of chemicals are being evaluated by using computational analyses of large primary databases held in public repositories and by identifying the most important findings in the burgeoning literature. These efforts are playing a central role in developing knowledge about the potential toxicity of chemicals and the causes of disease. These approaches, in combination with high-throughput approaches, could be used to support limited scope assessments or to augment robust traditional data-based assessments.
Developing innovative, targeted testing approaches that combine short-duration *in vivo* bioassays and HT approaches will provide even more robust information for testing and assessment.
Finally, a variety of new methods are addressing the formidable challenges of characterizing cumulative effects from exposure to multiple chemical and nonchemical stressors, susceptible subpopulations, and low-dose responses, primarily based on improving mechanistic understanding of adverse health effects.

Near-term efforts include developing additional prototypes for public input and peer review and providing more opportunities to solicit stakeholder comments and participation. The U.S. EPA is developing a verification process for new methods and data types that focuses on integrating the evidence into various decision contexts for use by risk assessors and considers the external validity of different models in terms of human relevance (U.S. EPA 2014). The goal is to increase confidence for using these new approaches in risk assessment. Significant scientific gaps identified in the completed and ongoing prototypes are helping guide future research plans. An overview of issues being considered is provided by Crawford-Brown (2013).

We anticipate the prototype demonstrations will help overcome the significant logistical and methodological challenges in interpreting and using these new data and methods in risk assessment. For now, major chemical assessments will continue to be driven primarily by traditional data but with increasing augmentation with the new types of data. The U.S. EPA risk managers and the risk assessment community at large will continue to be informed of the new tools and methods being developed with an emphasis on high-quality, human-relevant science and transparency. Historically difficult risk assessment questions that this new and emerging knowledge are likely to inform include *a*)Why do individual and specific populations respond differently to environmental exposures? *b*) How are children at greater risk for certain exposures and effects? *c*) What happens when people are exposed to mixtures that contain very low levels of individual chemicals, such as those commonly found in the environment? *d*) How do other environmental factors like preexisting health conditions alter the response to chemical exposures? These are just some of the issues that NexGen assessments will help address to improve the identification of safer chemicals and reduce risk from exposures to hazardous chemicals in the environment. A more detailed report is available (U.S. EPA 2014).

## Supplemental Material

(212 KB) PDF
