# Supplementary material for: The Next Generation of Risk Assessment Multi-Year Study—Highlights of Findings, Applications to Risk Assessment, and Future Directions
Source: Environ Health Perspect. 2016 Apr 19;124(11):1671–82. doi: 10.1289/EHP233 (PMC5089888; doi:10.1289/EHP233)
Supplement: (212 KB) PDF [file EHP233.s001.acco.pdf]

Environ Health Perspect
DOI: 10.1289/EHP233

Note to readers with disabilities: *EHP* strives to ensure that all journal content is accessible to all readers. However, some figures and Supplemental Material published in *EHP* articles may not conform to 508 standards due to the complexity of the information being presented. If you need assistance accessing journal content, please contact ehp508@niehs.nih.gov. Our staff will work with you to assess and meet your accessibility needs within 3 working days.

Supplemental Material

The Next Generation of Risk Assessment Multiyear Study—Highlights of Findings, Applications to Risk Assessment and Future Directions

Ila Cote, Melvin E. Andersen, Gerald T. Ankley, Stanley Barone, Linda S. Birnbaum, Kim Boekelheide, Frederic Y. Bois, Lyle D. Burgoon, Weihsueh A. Chiu, Douglas Crawford-Brown, Kevin M. Crofton, Michael DeVito, Robert B. Devlin, Stephen W. Edwards, Kathryn Z. Guyton, Dale Hattis, Richard S. Judson, Derek Knight, Daniel Krewski, Jason Lambert, Elizabeth Anne Maull, Donna Mendrick, Gregory M. Paoli, Chirag Jagdish Patel, Edward J. Perkins, Gerald Poje, Christopher J. Portier, Ivan Rusyn, Paul A. Schulte, Anton Simeonov, Martyn T. Smith, Kristina A. Thayer, Russell S. Thomas, Reuben Thomas, Raymond R. Tice, John J. Vandenberg, Daniel L. Villeneuve, Scott Wesselkamper, Maurice Whelan, Christine Whittaker, Ronald White, Menghang Xia, Carole Yauk, Lauren Zeise, Jay Zhao, and Robert S. DeWoskin

Table of Contents

Table S1. Governmental Partners

Table S2. An Illustrative Framework for Evidence Integration Focusing on New Data Types Used in the Prototypes

Table S1. Governmental Partners.

- Army Corps of Engineers
- California Environmental Protection Agency, Office of Environmental Health Hazard Assessment
- European Chemicals Agency
- European Joint Research Centre
- Health Canada
- L'Institut National de l'Environnement Industriel et des Risques
- U.S. Food and Drug Administration (National Center for Toxicological Research)
- U.S. Centers for Disease Control and Prevention, National Center for Environmental Health, and Agency for Toxic Substances and Disease Registry
- U.S. Department of Defense
- U.S. National Center for Advancing Translational Science
- U.S. National Institute of Environmental Health Sciences and National Toxicology Program
- U.S. National Institute for Occupational Safety and Health

Table S2: An Illustrative Framework for Evidence Integration Focusing on New Data Types Used in the Prototypes.

This causal determination framework illustrates how evidence integration and inferences about causality might be conducted using new data types. The left column summarizes the prototypes, the middle column presents evidence for causality exemplified by the prototypes, and the right column illustrates how such evidence might be integrated and weighed. To simplify the table, the prototypes with shared attributes are aggregated within a Tier. In general, data of equal quality data are weighed as follows: human data is preferred over non-human data, target tissue or cell data is preferred over non-target tissue or cell data, experimental systems with intact metabolism are preferred over systems without intact metabolism, *in situ*, tissue and primary cell culture are preferred over cell lines. It should be noted that all high quality data types have proved useful in evaluating risks. The Tier 3 of prototypes are unique in that they focus on known human health effects and well-documented public health risks in order to verify and anchor NexGen approaches. For the Tier 3 prototypes, the "Evidence Integration" column evaluates how successful new data types were in predicting known outcomes. While an example is provided here, our experience with evidence integration of various data types is limited and requires additional application experience.

Modifications of the Bradford-Hill criteria (e.g., consistency, coherence, biological plausibility) continued to be useful in the evaluation of new data types. As presented here, confidence in causality ranges from suggestive to likely, largely based on understanding the biological context in which new data types are embedded. "Likely" generally applies to the few cases for which the new data types are clearly tied to adverse outcomes through a combination of observational and experimental data, and include systems biology understanding. Biological context need not be chemical specific but also can be derived from disease/disorder-specific knowledge or from analogy with related chemicals. In practice, many new data types are anticipated to be "suggestive" for the near term and most appropriate for screening and prioritization and, perhaps, limited-scope assessments. Of note is that, contrary to traditional approaches, some new approaches can be used to estimate relative potencies to perturb important biologic processes as a potential surrogate for clearly identified hazards. We anticipate major assessments to be augmented by new data types but, for the near term, they will continue to be based on traditional data.

Prototypes		Evidence for Causality	Evidence Integration
**Tier 3**	**Molecular epidemiology and clinical studies:**<br>• Illustrated that new data types (when properly collected, analyzed, and reported) appear to provide results that are comparable to robust, traditional human data. These new data types could be used, when linked by mechanistic understanding, to:<br>(1) evaluate potential hazards posed by chemicals with no or limited traditional data, (2) augment traditional assessments, or (3) better	• **Specific pattern alterations in molecular events appear consistently and strongly associated with known intermediate events and known hazards at environmental exposure levels** (EPA 2013a, 2015; McCullough et al. 2014; McHale et al. 2009; McHale et al. 2012; Smith et al. 2011; Thomas et al. 2014). Data for tobacco smoke were reported in the Gene Expression Omnibus or ArrayExpress. Also see Cooper et al. 2013 and Hatzimichael and Crook 2013, Mattes et al, 2014).<br>• Exposure-dose was measured for benzene and ozone using urinary biomarkers and radiolabeled ozone, respectively (Hatch et al. 2014; McHale et al. 2009; Thomas et al. 2014). Tobacco-smoke exposures were self reported, substantially increasing uncertainty for exposure-dose-response characterization and highlighting the need for accurate exposure characterization (EPA 2014).<br>• Dose-dependent alterations are observed in concomitantly collected data for molecular events and adverse effects, in the range of	**Suggestive to likely:** Evidence is consistent, coherent, and biologically plausible that the observed molecular events are causally related to adverse effects. Implications are based on comparisons to robust traditional risk assessments:<br>• For benzene and ozone, identified molecular events are likely causally related to known adverse outcomes in a dose-dependent manner. Mechanistic links between molecular events, intermediate effects, and adverse outcomes are well understood. Pharmacologic intervention that blocks implicated pathways also blocks or ameliorates adverse effects. Similarly, measured polymorphism within identified pathways confers altered risks.

	inform traditional risk assessment issues, such as human variability and susceptibility, cross- species and *in vivo*-to-*in vitro* comparisons, cumulative risk, and low exposure-dose-response relationships.	environmental exposure (benzene and ozone). Some molecular pathways are altered at all concentrations; other molecular and toxicological effects emerge with increasing dose. Molecular patterns that occur consistently across all concentrations appear preferable as biomarkers (EPA 2013a; Hatch et al. 2014; McCullough et al. 2014; Thomas R et al. 2014).<br>• Pharmacological interventions have been shown to modify identified AOPs/AONs, and, concomitantly, the incidence or severity of the adverse outcomes (Cooper et al. 2013; Hatzimichael and Crook 2013; McCullough et al. 2014).	• In comparison, the molecular data for PAH are considered suggestive of a causal association between PAH and lung cancer due to lack of an observed exposure-dose-response relationship (likely due to uncertainties in exposure characterization), and data quality, analysis, and reporting limitations. Only about 8% of studies in Gene Expression Omnibus and ArrayExpress met study selection criteria.
**Tier 3 (continued)**		• Additional evidence for the involvement of specific pathways in disease is provided by identification of naturally occurring human gene variants in the AONs that alter susceptibility and risks (Cooper et al. 2013; Hatzimichael and Crook 2013; Moreno-Macias et al. 2013; Schlenk et al. 2008; Shen et al. 2011; Sillé et al. 2012; Smith et al. 2011; Vawda et al. 2014; Zhuo et al. 2012).<br>• AONs are also disrupted by other chemical and nonchemical stressors known to alter the incidence of the specific disease/disorder under consideration; thus, AONs provide a tool for evaluating cumulative risks based on mechanistic commonalities (IARC 2012; Smith et al. 2011; R Thomas et al. 2012).<br>• Supporting data are provided by multiple molecular epidemiology and clinical studies and chronic animal bioassays and coherence with other systems biology data (e.g., NIH BioSystems: acute myeloid leukemia, lung cancer (small cell, non-small cell); BaP and cancer: (EPA 2013b, a).<br>• Although species and *in vitro* differences exist, these examples provide consistent, coherent biologically plausible data linking specific omic alterations with specific diseases.	**Suggestive vs. likely:** In general, molecular data alone associated with adverse outcomes are expected to be only suggestive or are inadequate for causal determination. To rise to likely, the following are generally necessary:<br>• multiple, consistent, high-quality observational studies with similar results;<br>• understanding of the cascade of events between molecular events to adverse outcomes, and experimental evidence showing that reversal of pathway alterations blocks or ameliorates adverse outcome; or<br>• naturally occurring experiments where gene variants alter incidence or characteristic of disease.<br>Important variables such as experimental paradigm (e.g., *in vivo* vs. *in vitro*), cell type, tissue type, and species also require consideration. Suggestive data are likely to be most useful for hypothesis generation, discovery, screening and prioritization, and potential augmentation of traditional data.
**Tier 2**	**Knowledge mining and meta-analysis** prototype illustrated how large searchable databases can be used to identify, organize, integrate, and analyze existing data in an automated (computerized) fashion to	• **Knowledge mining and meta-analysis revealed associations between known exposures (biomonitoring) to several environmental agents and health effects** (e.g., metals and persistent organic chemical) with prediabetes/diabetes using the Centers for Disease Control and Prevention's National Health and Nutrition Assessment Examination Survey (NHANES). Human tissue biomarker and clinical outcome data are from the same individuals	**Suggestive to likely:** Associative data presented here are suggestive and generally most useful for hypothesis generation. Categorization could rise to likely with the types of supporting data noted above under Tier 1 "Suggestive vs. likely" or integration of consistent, coherent, biologically plausible data

	discover new insights into public health risks.	(EPA 2015; Patel et al. 2012; Patel et al. 2013).<br>• Supporting data are found in the literature (NIEHS 2015; Thayer et al. 2012).<br>• No systems biology context or Adverse Outcome Pathways (AOPs) or Adverse Outcome Networks (AONs) is available for data from NHANES. Information on biological context is available from the literature but currently not easily accessible in high- or medium-throughput approaches (Audouze et al. 2013; Inadera 2013).<br>• Also explored possible links among site-specific chemical exposures, ethnicity, genetic variants, and diabetes risks (Dimas et al. 2014; EPA 2015; Patel and Cullen 2012).	across data streams.
**Tier 2 (continued)**	**Short-duration *in vivo* exposure bioassays data use** in either alternative or rodent species is illustrated in two prototypes.	**Short-duration, *in vivo* exposure bioassays—alternative (nonmammalian) species prototype e.g. zebrafish developmental assay results, characterization of thyroid-specific mechanisms, and predictive modeling of complex dose-response phenomena.** Padilla et al. (2012) reported AC_{50}s (concentration at 50% of maximum activity) in a zebrafish developmental assay for 305 chemicals. Potencies for individual chemicals and chemical classes were shown to range over several orders of magnitude (1 nM–80 μM). For certain classes of chemicals, 80–100% in the class tested positive (embryo death or structural defects). Perkins et al. (2013) illustrated the use of alternative species to help articulate mechanisms showing an AON for thyroid disruption with example toxicants and alternative models applicable to both human and ecological hazard assessment. Also discussed are how predictive models coupled to mechanistic understanding can be used to better characterize dose-response (Sipes et al. 2011), circadian variations (Eisenberg et al. 2008), and exposure window-response relationships (DeWoskin et al. 2014). For more details on alternative species bioassays see Ankley and Gray (2013), Perkins et al. (2013), and Villeneuve et al. (2014).	**Suggestive to likely** within similar studies, coherent across different types of studies, and biologically plausible adverse phenotypic outcome data from nonmammalian, vertebrate species. Confidence is generally higher for evolutionarily conserved processes.<br><br>**Suggestive:** 1) Transcriptomic changes correlated to adverse outcomes studies and coupled to AOPs/AONs or high-content assays with measurable adverse outcomes (e.g., zebrafish developmental assay) generally have greater evidentiary weight than initiating event assays (e.g., transcriptomic assays). Some systems biology context is needed for limited-scope assessments for human risk (e.g., cross-species conservation, AOPs/AONs). Alternative species outcome data alone are sufficient for ecological risk assessment. Cross-species extrapolation and subchronic measurement of indicators introduces additional uncertainties as compared to human data discussed above. Reverse toxicokinetic models are needed to estimate equivalent human doses.
		**Short-duration, *in vivo* exposure bioassays—rodent prototype correlated transcriptomic alterations with adverse outcomes, as determined in traditional bioassays for 10 chemicals** (R Thomas et al. 2011; RS Thomas et al. 2012; RS Thomas et al. 2013). Consistency of the correlation between transcriptional changes and adverse effects across different exposure periods also was demonstrated (5 days to 13 weeks) Transcriptional changes appeared at somewhat lower	

		concentrations than traditional effects. Transcriptomic studies alone cannot predict specific hazards but might be useful in relatively ranking chemical potencies to induce biologic alterations that might precede adverse outcomes. In general, transcriptomic data require some biologic context (e.g., AOPs/AONs) to increase confidence of biologic significance.	
**Tier 1**	**Quantitative structure activity relationship (QSAR) and molecular docking models** are used to generate potency estimates and, with less confidence, hazards. Read-across is also considered (i.e., filling data gaps for data-poor chemicals by analogy with structurally related, more data-rich chemicals).	**QSAR models can predict chemical-specific toxicity values based on inherent chemical properties for some data-poor chemicals.**<br>• Models are developed based on chemical structures and known outcomes for data-rich chemicals (e.g., Goldsmith et al. 2012;Venkatapathy and Wang 2013; Wang et al. 2011; Wang et al. 2012a, 2012b).<br>• Organisation for Economic Co-operation and Development (OECD) is harmonizing international use of QSAR hazard models and read-across in the OECD QSAR toolbox (OECD 2014, 2015b, a). Also see EPA QSAR Guidance Document. (EPA 2012).<br>• Often, the consensus of a suite of appropriate models is the preferred approach.<br>• Often, models better predict potency than specific effects.<br>• Issues exist around characterizing the uncertainty in QSAR and related read-across approaches and in the transparency of some models. Also see Ball et al. (2014)and Patlewicz et al. (2013)	**Suggestive to likely:** TOPKAT Model predictions of potency when model is appropriate for chemicals evaluated; not generally predictive of dose-response for specific hazards; does generate a lowest-observed-adverse-effect level for a subset of the data-poor chemicals that meet confidence criteria. Additional OECD models and read-across can improve confidence in hazard characterization.
	**High-throughput, *in vitro* bioassays and virtual tissue models** are discussed.	**High-throughput *in vitro* assays** based on biological process disruptions are interpreted in systems biology and AOP/AON contexts and in association with adverse outcomes (Attene-Ramos et al. 2013; EPA 2015; Judson et al. 2014; Tice et al. 2013). Also see Huang et al. 2016. **Virtual tissue modeling** provides additional tools for evaluating data-limited chemicals (Knudsen et al. 2013).	**Suggestive:** When coupled with understanding of the AOPs/AONs. Could rise to likely with the types of supporting data noted above under "Suggestive to likely."

References

Ankley GT, Gray LE. 2013. Cross-species conservation of endocrine pathways: a critical analysis of tier 1 fish and rat screening assays with 12 model chemicals. Environ Toxicol Chem 32:1084–1087.

Attene-Ramos MS, Miller N, Huang R, Michael S, Itkin M, Kavlock RJ, et al. 2013. The Tox21 robotic platform for the assessment of environmental chemicals - from vision to reality. Drug Discov Today 18:716–273.

Audouze K, Brunak S, Grandjean P. 2013. A computational approach to chemical etiologies of diabetes. Sci Rep 3:2712; doi:10.1038/srep02712.

Ball N, Bartels M, Budinsky R, Klapacz J, Hays S, Kirman C, et al. 2014. The challenge of using read-across within the EU REACH regulatory framework; how much uncertainty is too much? Dipropylene glycol methyl ether acetate, an exemplary case study. Regul Toxicol Pharmacol 68:212–221.

Cooper WA, Lam DC, O'Toole SA, Minna JD. 2013. Molecular biology of lung cancer. J Thorac Dis 5 Suppl 5:S479–490.

DeWoskin RS, Knudsen TB, Shah I. 2014. Virtual models (vM). In: Encyclopedia of Toxicology, 3rd Edition, Volume 4 (Wexler P, ed). London, UK:Elsevier, Inc., 948–957.

DHHS (U.S. Department of Health and Human Services). 2014. The Health Consequences of Smoking - 50 Years of Progress. Rockville, MD:Office of the Surgeon General. Available: http://www.surgeongeneral.gov/library/reports/50-years-of-progress/exec-summary.pdf [accessed 28 April 2015].

Dimas AS, Lagou V, Barker A, Knowles JW, Magi R, Hivert MF, et al. 2014. Impact of type 2 diabetes susceptibility variants on quantitative glycemic traits reveals mechanistic heterogeneity. Diabetes 63:2158–2171.

Eisenberg MC, Samuels M, DiStefano JJ, 3rd. 2008. Extensions, validation, and clinical applications of a feedback control system simulator of the hypothalamo-pituitary-thyroid axis. Thyroid 18:1071–1085.

EPA (U.S. Environmental Protection Agency). 2012. Quantitative Structure Activity Relationship Guidance Document. Available: http://www.epa.gov/sites/production/files/2016-01/documents/qsar-guidance.pdf [accessed 07 March 2016].

EPA (U.S. Environmental Protection Agency). 2013a. Integrated Science Assessment of Ozone and Related Photochemical Oxidants (Final Report). EPA/600/R-10/076F. Washington, DC:U.S. EPA. Available: http://cfpub.epa.gov/ncea/isa/recordisplay.cfm?deid=247492 [accessed 28 Feb 2016].

EPA (U.S. Environmental Protection Agency). 2013b. IRIS Toxicological Review of Benzo[a]pyrene (Public Comment Draft). Washington, DC:EPA. Available: http://cfpub.epa.gov/ncea/iris_drafts/recordisplay.cfm?deid=66193 [accessed 18 Mar 2016].

EPA (U.S. Environmental Protection Agency). 2014. Next Generation Risk Assessment: Incorporation of Recent Advances in Molecular, Computational, and Systems Biology (Final Report). Washington, DC:U.S. EPA. Available: http://cfpub.epa.gov/ncea/risk/recordisplay.cfm?deid=286690 [accessed 07 March 2016].

Goldsmith MR, Peterson SD, Chang DT, Transue TR, Tornero-Velez R, Tan YM, et al. 2012. Informing mechanistic toxicology with computational molecular models. Methods Mol Biol 929:139–165.

Hatch GE, Duncan KE, Diaz-Sanchez D, Schmitt MT, Ghio AJ, Carraway MS, et al. 2014. Progress in assessing air pollutant risks from *in vitro* exposures: matching ozone dose and effect in human airway cells. Toxicol Sci 141:198–205.
Hatzimichael E, Crook T. 2013. Cancer epigenetics: New therapies and new challenges. J Drug Deliv 2013:529312; doi:10.1155/2013/529312:529312.
IARC. 2012. Benzene. IARC Monogr Eval Carcinog Risk Hum 100F:249–294.
Huang R, Xia M, Sakamuru S, Zhao J, Shahane SA, Attene-Ramos M, et al. 2016. Modelling the Tox21 10 K chemical profiles for in vivo toxicity prediction and mechanism characterization. Nat Commun. 26;7:10425.
Inadera H. 2013. Developmental origins of obesity and type 2 diabetes: molecular aspects and role of chemicals. Environ Health Prev Med 18:185–197.
Judson R, Houck K, Martin M, Knudsen TB, Thomas RS, Sipes N, et al. 2014. *In vitro* and modelling approaches to risk assessment from the U.S. Environmental Protection Agency ToxCast programme. Basic Clin Pharmacol Toxicol 115:69–76.
Knudsen T, Martin M, Chandler K, Kleinstreuer N, Judson R, Sipes N. 2013. Predictive models and computational toxicology. Methods Mol Biol 947:343–374.
Mattes W, Yang X, Orr MS, Richter P, Mendrick DL. 2014. Biomarkers of tobacco smoke exposure. Adv Clin Chem. 67:1-45.
McCullough SD, Duncan KE, Swanton SM, Dailey LA, Diaz-Sanchez D, Devlin RB. 2014. Ozone induces a pro-inflammatory response in primary human bronchial epithelial cells through mitogen-activated protein kinase activation without nuclear factor-κB activation. Am J Respir Cell Mol Biol 51:426–435.
McHale CM, Zhang L, Lan Q, Li G, Hubbard AE, Forrest MS, et al. 2009. Changes in the peripheral blood transcriptome associated with occupational benzene exposure identified by cross-comparison on two microarray platforms. Genomics 93:343–349.
McHale CM, Zhang L, Smith MT. 2012. Current understanding of the mechanism of benzene-induced leukemia in humans: Implications for risk assessment. Carcinogenesis 33:240–252.
Moreno-Macias H, Dockery DW, Schwartz J, Gold DR, Laird NM, Sienra-Monge JJ, et al. 2013. Ozone exposure, vitamin C intake, and genetic susceptibility of asthmatic children in Mexico City: a cohort study. Respir Res 14:14; doi:10.1186/1465-9921-14-14.
NIEHS (National Institute of Environmental Health Sciences). 2015. Comparative Toxicogenomic Database (CTD)™. Available: http://ctdbase.org/ [accessed 28 April 2015].
NRC (National Research Council). 2014. Review of EPA's Integrated Risk Information System (IRIS) Process. Washington, DC:The National Academies Press. Available: http://www.nap.edu/catalog.php?record_id=18764 [accessed 01 March 2016].
OECD (Organisation for Economic Cooperation and Development). 2016. The OECD QSAR Toolbox. Version 3.35. Available: http://www.oecd.org/env/chemicalsafetyandbiosafety/assessmentofchemicals/theoecdqsartoolbox.htm [accessed 01 March 2016].
OECD (Organisation for Economic Cooperation and Development). 2015a. Grouping of Chemicals: Chemical Categories and Read-Across. Available: http://www.oecd.org/chemicalsafety/risk-assessment/groupingofchemicalschemicalcategoriesandread-across.htm [accessed 10 March 2016].

OECD (Organisation for Economic Cooperation and Development). 2015b. Other Activities on Molecular Screening and Toxicogenomics. Available: http://www.oecd.org/env/ehs/testing/toxicogenomics.htm [accessed 10 March 2016].
Padilla S, Corum D, Padnos B, Hunter DL, Beam A, Houck KA, et al. 2012. Zebrafish developmental screening of the ToxCast™ Phase I chemical library. Reprod Toxicol 33:174–187.
Patel CJ, Chen R, Butte AJ. 2012. Data-driven integration of epidemiological and toxicological data to select candidate interacting genes and environmental factors in association with disease. Bioinformatics 28:i121–126.
Patel CJ, Chen R, Kodama K, Loannidis JP, Butte AJ. 2013. Systematic identification of interaction effects between genome-and environment-wide associations in type 2 diabetes mellitus. Hum Genet 132:495–508.
Patel CJ, Cullen MR. 2012. Genetic variability in molecular responses to chemical exposure. EXS 101:437–457.
Patlewicz G, Ball N, Booth ED, Hulzebos E, Zvinavashe E, Hennes C. 2013. Use of category approaches, read-across and (Q)SAR: general considerations. Regul Toxicol Pharmacol 67:1–12.
Perkins EJ, Ankley GT, Crofton KM, Garcia-Reyero N, Lalone CA, Johnson MS, et al. 2013. Current perspectives on the use of alternative species in human health and ecological risk assessments. Environ Health Perspect 121:1002–1010.
Rhomberg LR, Goodman JE, Bailey LA, Prueitt RL, Beck NB, Bevan C, et al. 2013. A survey of frameworks for best practices in weight-of-evidence analyses. Crit Rev Toxicol 43:753–784.
Schlenk RF, Dohner K, Krauter J, Frohling S, Corbacioglu A, Bullinger L, et al. 2008. Mutations and treatment outcome in cytogenetically normal acute myeloid leukemia. N Engl J Med 358:1909–1918.
Shen M, Zhang L, Lee KM, Vermeulen R, Hosgood HD, Li G, et al. 2011. Polymorphisms in genes involved in innate immunity and susceptibility to benzene-induced hematotoxicity. Exp Mol Med 43:374–378.
Sillé FC, Thomas R, Smith MT, Conde L, Skibola CF. 2012. Post-GWAS functional characterization of susceptibility variants for chronic lymphocytic leukemia. PloS One 7:e29632; doi:10.1371/journal.pone.0029632:e29632.
Sipes NS, Padilla S, Knudsen TB. 2011. Zebrafish: as an integrative model for twenty-first century toxicity testing. Birth Defects Res C Embryo Today 93:256–267.
Smith MT, Zhang L, McHale CM, Skibola CF, Rappaport SM. 2011. Benzene, the exposome and future investigations of leukemia etiology. Chem Biol Interact 192:155–159.
Thayer KA, Heindel JJ, Bucher JR, Gallo MA. 2012. Role of environmental chemicals in diabetes and obesity: a National Toxicology Program workshop review. Environ Health Perspect 120:779–789.
Thomas R, Hubbard A, McHale CM, Zhang L, Rappaport SM, Rothman N, et al. 2014. Characterization of changes in gene expression and biochemical pathways at low levels of benzene exposure. PloS One 9:e91828; doi:10.1371/journal.pone.0091828.
Thomas RS, Clewell HJ, 3rd, Allen BC, Wesselkamper SC, Wang NC, Lambert JC, et al. 2011. Application of transcriptional benchmark dose values in quantitative cancer and noncancer risk assessment. Toxicol Sci 120:194–205.
Thomas RS, Clewell HJ, 3rd, Allen BC, Yang L, Healy E, Andersen ME. 2012. Integrating pathway-based transcriptomic data into quantitative chemical risk assessment: a five chemical case study. Mutat Res 746:135–143.

Thomas RS, Wesselkamper SC, Wang NC, Zhao QJ, Petersen DD, Lambert JC, et al. 2013. Temporal concordance between apical and transcriptional points of departure for chemical risk assessment. Toxicol Sci 134:180–194.
Tice RR, Austin CP, Kavlock RJ, Bucher JR. 2013. Improving the human hazard characterization of chemicals: a Tox21 update. Environ Health Perspect 121:756–765.
Vawda S, Mansour R, Takeda A, Funnell P, Kerry S, Mudway I, et al. 2014. Associations between inflammatory and immune response genes and adverse respiratory outcomes following exposure to outdoor air pollution: a HuGE systematic review. Am J Epidemiol 179:432–442.
Venkatapathy R, Wang NC. 2013. Developmental toxicity prediction. Methods Mol Biol 930:305–340.
Villeneuve D, Volz DC, Embry MR, Ankley GT, Belanger SE, Leonard M, et al. 2014. Investigating alternatives to the fish early-life stage test: a strategy for discovering and annotating adverse outcome pathways for early fish development. Environ Toxicol Chem 33:158–169.
Wang N, Venkatapathy R, Bruce RM, Moudgal C. 2011. Development of quantitative structure-activity relationship (QSAR) models to predict the carcinogenic potency of chemicals. II. Using oral slope factor as a measure of carcinogenic potency. Regul Toxicol Pharmacol 59:215–226.
Wang N, Rice GE, Teuschler LK, Colman J, Yang RS. 2012a. An in silico approach for evaluating a fraction-based, risk assessment method for total petroleum hydrocarbon mixtures. J Toxicol 2012:410143.
Wang N, Zhao JQ, Wesselkamper SC, Lambert JC, Petersen D, Hess-Wilson JK. 2012b. Application of computational toxicological approaches in human health risk assessment. I. A tiered surrogate approach. Regul Toxicol Pharmacol 63:10–19.
Zhuo W, Zhang L, Zhu B, Qiu Z, Chen Z. 2012. Association between CYP1A1 Ile462Val variation and acute leukemia risk: meta-analyses including 2164 cases and 4160 controls. PloS One 7:e46974; doi:10.1371/journal.pone.0046974.
